# Dose-Response-Relationship between Number of Laser Burns and IOP Reduction in Cyclophotocoagulation: An Animal Study

**DOI:** 10.1155/2014/983102

**Published:** 2014-06-29

**Authors:** Lars Wagenfeld, Hendrik Schwarzer, Gernot Roessler, Maren Klemm, Christos Skevas, Gisbert Richard, Oliver Zeitz

**Affiliations:** ^1^Department of Ophthalmology, University Medical-Center Hamburg-Eppendorf, 20246 Hamburg, Germany; ^2^Department of Ophthalmology, RWTH Aachen University, 52074 Aachen, Germany; ^3^Global Clinical Development, Bayer HealthCare AG, 13353 Berlin, Germany

## Abstract

*Purpose*. The relationship between number of laser burns of cyclophotocoagulation (CPC) and intraocular pressure (IOP) reduction is unknown. This animal model was established to reveal a possible dose-response-relationship between the number of applied laser burns and the IOP lowering effect. *Methods*. 30 chinchilla bastard rabbits were divided into 5 groups and treated with either 1, 5, 10, 20, or 30 CPC burns, respectively. IOP was followed up for 1 week. IOP reduction of a single 30-burn treatment was compared with a fractionated treatment (three sessions; one week in between; 10 burns/session). *Results*. IOP reduction increases nonlinearly with the number of CPC burns (max. −6.1 ± 1.4 mmHg). Fractionated treatment shows similar IOP reduction with less complications and more constant results compared to single session treatment. *Conclusions*. The study reveals a complex relationship between IOP reduction and the number of CPC burns. Fractionated CPC gives comparable IOP reduction at a higher degree of safety.

## 1. Background

In glaucoma therapy, IOP reduction, medical or surgical, is the most important goal [1, 2]. Surgical approaches include filtrating and cyclodestructive procedures. Among the latter, cyclophotocoagulation (CPC) is a frequently chosen treatment [3]. In CPC, laser burns are applied to the ciliary epithelium, typically in a transscleral approach [4]. Because this laser-surgical procedure is very easy and fast to be performed, many glaucoma specialists use it on a large scale [5, 6]. Compared to other cyclodestructive procedures such as cyclocryotherapy, side effects are rarer and the whole procedure is less traumatic [7]. Nevertheless, severe complications such as hypotonia and phthisis bulbi can be seen after CPC treatment [8, 9]. On an empirical basis, the higher the number of laser burns applied, the more likely it is that these postoperative courses could occur ([8, 10] and own unpublished observations). The main problem in planning the optimal number of laser burns for CPC treatment is that the IOP reduction induced by each laser burn is unpredictable [10, 11]. Investigating this problem on a clinical level is limited by ethical considerations as well as by methodological difficulties. Patients undergoing CPC are mostly pretreated with antiglaucomatous drugs. After surgery, they are usually no longer on medication. Thus, the IOP levels before and after the intervention cannot be compared adequately. Therefore, we decided to set up an animal model for CPC to study the basic IOP-lowering effects in a very defined setting. To achieve the most stable environment for the experiments, healthy animals were used. Two problems were addressed. In the first set of experiments, the relationship between IOP reduction and number of laser burns as well as the IOP reduction per laser burn was determined. In the second set of experiments, fractionation of the CPC treatment was studied. We hypothesised that for a certain number of laser burns the efficiency of CPC would be similar whether burns were applied in one session or fractionated to multiple sessions.

## 2. Methods

### 2.1. Animals and CPC Treatment Procedure

The experiments were assessed and approved in advance by the local ethics committee of the medical council of Hamburg (authorization 13/05). All experiments were conducted in adherence to the ARVO statement for the use of animals in ophthalmic and vision research. A total of thirty healthy Chinchilla bastard rabbits were included in this study. These animals were distributed into five groups and treated with 1, 5, 10, 20, or 30 laser burns. Rabbits receiving 10 laser burns in the first session were treated in two more sessions with 10 laser burns per treatment at intervals of 1 week. These rabbits were compared to rabbits that were treated with 30 laser burns in the first session.

The treatment was performed under general anaesthesia with intravenously administered propofol and analgesia with locally applied lidocaine. An Iris Medical diode laser (810 nm) with a standard Iris Medical cyclophotocoagulation laser probe was used for all treatments. The probe was placed perpendicularly on the conjunctiva so that the burns were placed in a distance of 1-1.5 mm behind the limbus corresponding to the ciliary body. Before the treatment, the ciliary body was identified by transscleral diaphanoscopy. For application of up to 10 laser burns, all laser burns were placed in the lower hemisphere; when higher numbers were used, laser burns were distributed over the complete 360° circumference. While administering the laser burns, pop effects were minimised by varying the exposure time between 1.5 and 2.0 s, which results in an energy of 3-4 J.

### 2.2. Measurement of IOP

IOP was measured using a Tonopen XL under local anaesthesia induced with 1% oxybuprocaine eye drops. Each IOP measurement consisted of three single measures that were averaged and treated statistically as a measured variable.

### 2.3. Relationship between Number of Laser Burns and IOP Reduction

To investigate the relationship between the number of applied laser burns and the achieved IOP reduction, IOP was measured 1 day before and 1 week after CPC treatment. The difference between the treated and the untreated eye (ΔIOP) was evaluated. The effect of CPC on ΔIOP was analysed by a paired, double-sided* t*-test. ΔIOP was correlated with the number of laser burns by Pearson's correlation coefficient using SPSS 10.0.

### 2.4. Effect of Fractionation of CPC on IOP Reduction

ΔIOP was tracked for 7 weeks in both the group that received 30 laser burns in a single session and the group receiving the 30 burns fractionated over three sessions. Differences between groups were evaluated using an unpaired, double-sided* t*-test.

### 2.5. General Statistics

All measures are presented as mean ± standard error of means (SEM); *P* < 0.05 was considered to be statistically significant.

### 2.6. Statements of Ethics

We certify that all applicable institutional and governmental regulations concerning the ethical use of animals were followed during this research.

## 3. Results

**Figure 1:**
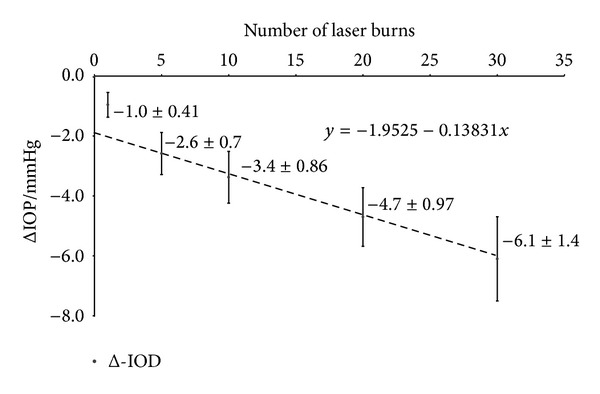
The figure shows the IOP differences between treated and control eye in relation to the number of laser burns applied. The dashed line and the equation are the result of a linear curve fit for ΔIOP at five or more laser burns (for interpretation, refer to the text).

In a first set of experiments, rabbits were treated with 1 to 30 laser burns. The ΔIOP was determined before and 1 week after treatment. Before treatment, ΔIOP was virtually zero; however, it increased up to 6.1 ± 1.4 mmHg after treatment with 30 burns. Even a single laser burn had a slight IOP-reducing effect. The relationship between the number of burns and the ΔIOP is shown quantitatively in Table 1 and Figure 1. The calculated coefficient of correlation between ΔIOP and the number of laser burns was 0.65; *P* < 0.01.

The IOP reduction per laser burn is also shown in Table 1. This decreased as more laser burns were applied.

In the second part of the study, six rabbits were treated with 30 laser burns in a single session; six other rabbits received a total of 30 burns fractionated over three sessions, 1 week apart, with 10 laser burns applied at each session. The ΔIOP was monitored for 7 weeks. Comparison of ΔIOP between both groups using an unpaired* t*-test showed a significant difference 1 week after the first treatment. After the second treatment in the fractionated group, differences were no longer significant. Quantitative results and *P* values are shown in Table 2. In the group receiving all burns in a single session, four of the six rabbits had an intraocular haemorrhage on the first postoperative day. In one rabbit, this haemorrhage led to an irreversible ocular hypertension with an IOP >50 mmHg. This animal was excluded from the statistical analysis. In the fractionated group, only one animal had a rapidly resorbing anterior chamber bleeding after the first treatment.

In an additional analysis, the number of animals reaching a certain target IOP reduction was counted. The result is summarised in Table 3. Briefly, in the fractionated group, more rabbits seemed to reach a higher target IOP reduction.

## 4. Discussion

The first part of the study addressed the relationship between the number of applied laser burns and IOP reductive effect. At first glance, the obtained results do not follow any simple mathematical rules. The relationship obtained from the present results shows a nearly linear characteristic for higher number of laser burns (dashed line in Figure 1) but deviates from this linear relation at less than five laser burns. This implies a combination of at least two effects. Conventionally, the mechanism behind the IOP reduction induced by CPC has been explained through the loss of secretory ciliary epithelium [12, 13], which can be found soon after CPC treatment [14]. If no compensatory mechanisms were involved, each laser burn would result in a more constant drop of secreted volume. Taking several facts into account, including sclera compliance, Friedland published a formula for a change of pressure induced by certain changes of intraocular volumes:

(1) $$\begin{array}{c} \Delta p\left(t\right)\approx \frac{2\alpha p}{r}\Delta v\left(t\right), \end{array}$$

where Δ*p*(*t*) = change in pressure (mmHg), *p* = pressure (mmHg), *α* = elastic constant (cm^−2^), Δ*v*(*t*) = change in volume (*μ*L), and *r* = radius (cm) [15]. Within physiological limits, this relationship is linear. So, the IOP reduction after application of five and more laser burns may be explained by reduced secretory power of the ciliary pigment epithelium as it can be described by a straight line. But, interestingly, this straight line has a negative offset of *≈*−1.95 mmHg according to the equation of the linear fit in Figure 1. This means that the IOP-reducing effect achieved by reduced aqueous humor secretion is enhanced by 1.95 mmHg, which explains the paradox strong IOP-reducing effect of a single laser burn. We hypothesize that this enhancing effect is due to the secretion of inflammatory mediators, which could be, for example, prostaglandins. It is known that a laser treatment such as CPC provokes an inflammatory response [16, 17], and it is well established that prostaglandins are a mediator of inflammation [18]. In addition, some prostaglandins have been shown to lower IOP effectively [19].

The second part of the study was dedicated to comparing fractionated* versus* nonfractionated treatment. The results indicate a comparable IOP lowering effect for both treatments, but with apparently less frequent and less severe complications associated with the fractionated treatment. In accordance with data from clinical trials, severe complications occurred only in the nonfractionated, single-session group in the present study [3, 20, 21]. The 30 burns that were applied to this group were, with respect to the size of the rabbit eye, an overtreatment, with burns being placed over the entire circumference of the ciliary body. In this group, two-thirds of the animals had intraocular haemorrhages. In this context, it has to be pointed out that the increased complication frequency was not associated with any advantage regarding the pressure reduction. In contrast to clinical reports, in the presented animal series, no case of hypotony was observed. Walland et al., who compared IOP reduction of a standard protocol for full- and half-dose-treated patients, reported hypotony in 18% of the full-dose-treated eyes [8].

The risk profile of CPC leads to a controversy as to which patients should be offered CPC. CPC is discussed as an appropriate alternative for the therapy for end-stage glaucoma patients [5, 6]. To minimise the risk, in recent years, several glaucoma specialists have reduced the number of laser burns applied per session. In addition, it has been postulated that the treatment can be repeated if the target pressure is not reached [5, 6, 22, 23]. Taken together, these reports imply that a lower number of laser burns have a more favourable risk profile and that the IOP lowering effect of multiple treatment sessions may be additive. The present study provides an experimental basis for these clinical experiences. Comparing the IOP reduction of the single treatments in the fractionated group, the same number of laser burns has a higher effect in the first treatments (Table 2, weeks 1–3 in the nonfractionated group). This is a further indicator that there is no simple dose-response-relationship.

It is difficult to compare our results with previous work directly since these studies particularly address the laser parameters for a single laser burn, but not the relationship between the number of laser burns and mode of application [24]. Other studies compare different technical systems for CPC [16, 25]. Therefore, the presented data put a new perspective and show new information on the effects of CPC treatment.

When drawing conclusions from the presented data, one has to take into account the fact that the results were obtained from healthy animals. On the other hand, animal models mimicking the dysregulated IOP in humans with glaucoma are rare. Most animal models of glaucoma are ocular hypertension models and are generated by surgical occlusion of aqueous outflow. The course and duration of ocular hypertension are difficult to predict and surgical side effects may interfere with the CPC effect, particularly the hypothesized inflammatory component. Therefore, we feel that experiments with healthy animals give the most conclusive results since interfering effects and model immanent uncertainties are minimized.

The study of course is limited by the obvious differences between physiology and anatomy of the rabbit and the human eye, but investigating this issue in humans is limited by considerations made in the introduction of this paper. Due to governmental regulations concerning the ethical use of animals, rabbits were the biggest animals granted for this study. However, the anatomy of the rabbit eye has been investigated in 1929 by Davis and has shown that the ciliary processes encroach far forward on the posterior surface of the iris [26]. Histological findings of Schubert and Federman in 1989 showed that after cyclophotocoagulation the ciliary body processes were flattened and covered by superficial fibrous tissue attached to the lens equator [27]. In different studies, from 1985 to 2001, several authors established animal models using rabbits and choosing a limbal distance of 1-1.2 mm for the application of the laser burns [28–31]. In the histological workup, thereafter, a significant thermal destruction of the ciliary body with its processes could be seen and a significant lowering of the IOP by cyclophotocoagulation was proven. However, in none of these approaches, a clear correlation of pars plicata destruction and a IOD lowering effect could be shown. Each of the single possible components in IOP lowering by cyclophotocoagulation, such as epithelial ablation, vascular ablation, increased uveoscleral outflow, inflammation, and suprachoroidal cleft formation, seems to play a certain role [13, 29, 32, 33].

## 5. Conclusions

Our experiments show that, in the short term, the first laser burns applied appear to be the most effective. This could be due to an excretion of, for example, prostaglandins due to an inflammatory effect. The more laser burns applied, the more important the cyclodestructive component of the IOP-lowering effect apparently becomes. This combination of two mechanisms results in a complex relationship between applied laser burns and IOP lowering effect. This makes it unlikely that a general form of nomogram for CPC can be set up. Separating the CPC into several sessions, as proposed in the fractionated CPC concept, might solve this problem since it provides comparable IOP reduction at a higher degree of safety.

## Figures and Tables

**Table 1:** IOP differences between treated and control eye (ΔIOP in mmHg), before and after treatment, and calculated IOP reduction per laser burn. A negative difference means that the IOP of the treated eye is lower than that of the control eye.

Laser burns	1	5	10	20	30
Before treatment	0.1 ± 0.2	0 ± 0.3	−0.2 ± 0.3	−0.3 ± 0.2	−0.3 ± 0.2
After treatment	−1.0 ± 0.4	−2.6 ± 0.6	−3.4 ± 0.8	−4.7 ± 1.0	−6.1 ± 1.4
*P* value	0.02	<0.01	0.01	0.01	0.01
IOP reduction per laser burn	0.96 ± 0.37	0.52 ± 0.13	0.34 ± 0.08	0.24 ± 0.05	0.20 ± 0.05

**Table 2:** ΔIOP between the treated and untreated eye in fractionated and nonfractionated groups after 7 weeks of follow-up (*P* values in parentheses indicate a statistical comparison with preop; *P* in the last column indicates statistical comparison between fractionated and nonfractionated groups).

Time	Fractionated	Nonfractionated	*P*
Preop	0.5 ± 0.4	0.3 ± 0.2	0.51
Week 1	−2.8 ± 1.2 (*P* = 0.03)	−6.1 ± 1.4 (*P* = 0.01)	0.05
Week 2	−6.7 ± 1.1 (*P* ≤ 0.01)	−3.0 ± 3.3 (*P* = 0.37)	0.93
Week 3	−7.0 ± 0.8 (*P* ≤ 0.01)	−3.6 ± 3.2 (*P* = 0.33)	0.79
Week 4	−5.2 ± 0.6 (*P* ≤ 0.01)	−5.6 ± 0.9 (*P* ≤ 0.01)	0.56
Week 5	−4.2 ± 0.5 (*P* ≤ 0.01)	−6.8 ± 1.5 (*P* = 0.02)	0.28
Week 6	−3.0 ± 0.6 (*P* ≤ 0.01)	−5.6 ± 1.4 (*P* = 0.03)	0.26
Week 7	−3.0 ± 0.3 (*P* ≤ 0.01)	−3.8 ± 0.9 (*P* = 0.02)	0.66

**Table 3:** Number of rabbits reaching a hypothetic target IOP reduction.

Target IOP reduction	Fractionated treatment			Nonfractionated treatment
	10 burns	+10 burns	+10 burns	30 burns
3 mmHg	4/6	6/6	6/6	5/5
4 mmHg	2/6	5/6	6/6	4/5
5 mmHg	1/6	4/6	5/6	3/5
6 mmHg	1/6	3/6	5/6	2/5
